# COMT but Not 5HTTLPR Gene Is Associated with Depression in First-Episode Psychosis: The Role of Stressful Life Events

**DOI:** 10.3390/genes14020350

**Published:** 2023-01-29

**Authors:** Sarah Tosato, Chiara Bonetto, Katia De Santi, Antonio Lasalvia, Massimo Gennarelli, Doriana Cristofalo, Mariaelena Bertani, Mirella Ruggeri, on behalf of the PICOS-Veneto Group

**Affiliations:** 1 Department of Neuroscience, Biomedicine and Movement Sciences, Section of Psychiatry, University of Verona, 37134 Verona, Italy; 2 Azienda Ospedaliera Universitaria Integrata, Unit of Psychiatry B, 37134 Verona, Italy; 3 Department of Molecular and Translational Medicine, University of Brescia, 25121 Brescia, Italy; 4 Genetics Unit, IRCCS Istituto Centro San Giovanni Di Dio Fatebenefratelli, 25121 Brescia, Italy

**Keywords:** COMT, 5-HTTLPR, stressful life events, depression, first-episode psychosis

## Abstract

Serotonergic and dopaminergic systems are involved in the regulation of mood and reactivity to psychological stress. This study explores, in a sample of first episode psychosis (FEP) patients, whether more severe depressive symptoms were found in those who: (1) experienced a major stressful event in the 6 months preceding illness onset; and (2) were homozygous for the COMT Val158 allele or carrying the S allele of 5-HTTLPR. A total of 186 FEP patients recruited were assessed using the Hamilton Rating Scale for Depression (HAMD) for depressive symptoms. Stressful life events (SLEs) were collected by the List of Events Scale. The genotypes of 5-HTTLPR, rs25531, and COMT Val158 Met were performed. It has been found that higher levels of depression is associated with the presence of SLEs (*p* = 0.019) and with COMT Val158 allele homozygosity (*p* = 0.029), but not with carrying the S allele of 5-HTTLPR. The COMT gene moderates the association between depression and SLEs as Val158 allele homozygote patients experiencing SLEs had the highest level of depressive symptoms compared to the others (*p* = 0.002). The present study provides initial evidence for an effect of the COMT Val158 homozygosity and severe stressful life events on the severity of depressive symptoms in first episode psychosis.

## 1. Introduction

Depressive symptoms can occur during both the acute or post-acute phase of first episode psychosis and have been associated with adverse outcomes including increased risk of relapse and hospitalization and poor quality of life [1]. Evidence indicates that depressive disorder is highly prevalent in first episode psychosis since it affects at least one-quarter of patients, confirming depressive psychopathology as an inextricable symptom domain of psychosis [2]. 

Several studies have shown that a dysfunction of the serotonergic and dopaminergic systems exerts a major influence on the brain circuits involved in the regulation of mood and reactivity to psychological stress [3,4]. Genetic variants in both the serotonin transporter gene (SLC6A4, 5-HTT, or SERT) and in the catechol-O-methyltransferase (COMT) gene have been found to contribute to the pathophysiology of depressive disorder [5,6]. Regarding the first, the serotonin transporter gene harbors a functional 43 bp insertion/deletion polymorphism (5-HTTLPR), which yields a short (S) and a long (L) allele [7]. The S allele is associated with a nearly 50% reduction in the basal 5-HTT activity in vitro [8] and, consequently, it has also been associated with the development of depression [5] in subjects affected by chronic schizophrenia [9]. It has also been found that a polymorphism (rs25531) located near the 5-HTTLPR locus consists of an A to G substitution and modulates the effect of 5-HTTLPR on transcriptional efficacy [5]. The Single Nucleotide Polymorphism (SNP) (rs25531) divided L into LA, with high 5-HTT transcriptional activity, and LG, which is functionally equivalent to the low-expressing S allele as a risk factor of depression [10]. Therefore, the modulation of the 5-HTTLPR by rs25531 results in haplotypes with high (LA) or low (LG, SA, or SG) transcriptional efficacy [11,12]. Among the genes modulating dopamine transmission, the COMT gene harbors the Val158Met polymorphism (rs4680), which influences brain morphology and functional connectivity in psychosis [13]. Furthermore, the Val158 allele is associated with a high enzymatic activity, leading to a reduction in dopamine in the prefrontal cortex (PFC) [14]. The Val158 allele has been related to depressive disorder [6], while there is no compelling evidence of association in schizophrenia [15,16]. 

Furthermore, it is well-established that stressful life events (SLEs) often occur shortly before the onset of psychosis [17,18] and of depression [19,20], contributing to both disorders by “activating’ a genetic vulnerability [21]. There is evidence reporting that individuals with the S/S genotype of the 5-HTTLPR polymorphism are more sensitive to stressful life events than those with the S/L or L/L genotype [22]. Regarding dopamine, the Met allele has been found to exert an alleviative effect on depression in children with institutional care experience [23]. This was confirmed by a study finding that adolescents with the Val/Val genotype showed more depressive symptoms when they experienced harsher parenting [24].

Based on these results, the present study aims, in a sample of first episode psychosis patients, to explore whether a higher depression at the onset was found in those who: (1) had at least one major stressful event in the previous 6 months; and (2) were homozygous for the COMT Val^158^ allele or carrying the S allele of 5-HTTLPR. Moreover, a possible effect of events and genotypes on depression was explored, specifically, whether patients who had at least a major stressful event in the previous 6 months and were homozygous for the COMT Val^158^ allele or carrying the S allele of 5-HTTLPR showed the highest level of depression.

## 2. Methods

### 2.1. Participants

Based on the WHO 10-Country study [25], first episode psychosis subjects accessing Community Mental Health Centers (CMHCs) in the Veneto Region (Italy) were asked to be recruited and assessed. Patients provided written informed consent. The study was approved by the Ethics Committee of the coordinating Center and by the local Ethics Committees of participating sites. Details on the study design and recruitment have been reported elsewhere [26,27,28].

Briefly, patients were recruited if (1) their age was between 18 and 54 years; (2) they were resident within the catchment areas; (3) they contacted the CMHCs located in the catchment areas for the first time in their life for at least one symptom among hallucinations, delusions, speech disorder, psychomotor disorder, bizarre, or inappropriate behavior; or two symptoms among loss of interest, social withdrawal, severe excitement, purposeless destructiveness, overwhelming fear, or self-neglect. Patients were excluded if they were under antipsychotic medication for more than three months, if they were affected by a mental disorder due to general medical condition, or by a moderate–severe intellectual disability or by psychiatric diagnosis other than ICD-10 psychosis.

### 2.2. Measures

The Positive and Negative Symptoms Scale (PANSS) [29] for general psychopathology and the 21 items Hamilton Rating Scale for Depression (HAMD) [30] for depressive symptoms were used to assess the patients. The Item Group Checklist (IGC) of the Schedules for Clinical Assessment in Neuropsychiatry (SCAN) [31] was applied to confirm diagnosis after six months. 

The Life Events Scale [32] was used to assess stressful events that had occurred in the previous 6 months. The severity of each event was assessed by the Holmes–Rahe Life Stress Inventory [33]. As conducted previously [18,34], ‘severe stressful life events’ (SLEs) were used in the analyses (i.e., death of a family member, sexual or physical abuse, being accused of having committed a crime, sentence of imprisonment, being exposed to war and natural catastrophes, family breakdown, being removed from home, sentimental breakdown, and severe physical illness). 

### 2.3. Genotyping 

EDTA-containing tubes were used to collect venous blood (15 mL) from each subject, and a commercial kit (ABgene, Blenheim Road, Epson, Surrey, UK) was used to extract the DNA from peripheral blood

The genotypes of 5-HTTLPR and rs25531 were performed according to Bonvicini et al. [35]. PCR products were directly used for 5HTTLPR genotyping. PCR products (15 μL) were digested with the MspI enzyme (Fermentas) to confirm the 5HTTLPR/rs25531 genotype. The MspI enzyme recognizes and cuts a C|CGG sequence, leading to the following fragments: LA = (325 + 62 + 33) bp, LG = (174 + 150 + 62 + 33) bp, SA = (281 + 62 + 33) bp, and SG = (150 + 131 + 62 + 33) bp. The digestion was performed overnight at 37 °C and analyzed via agarose gel electrophoresis with an agarose concentration of 3.5%. Genotypes were determined by two independent blinded investigators. A TaqMan^®^ SNP Genotyping Assay (Applied Biosystems, Assay ID C_25746809_50), according to the manufacturer’s instructions, was used to determine the COMT Val158Met genotype. Endpoint analyses were performed by anABI7900 DNA analyzer (Applied Biosystems, Foster City, CA, USA), and the genetic analysis criteria were as follows: (i) genotypes formed three distinct clusters; (ii) water controls were negative; (iii) the number of callable genotypes was >90%; and (iv) minor allele frequency was greater than 2%. Furthermore, inter-plate and intra-plate duplicate testing of known DNAs was conducted.

### 2.4. Statistical Analyses 

Each categorical variable was described by percentage distribution and each continuous variable by the mean and SD (standard deviation). The association between categorical variables was estimated by the Chi-square test, while differences in the mean scores of continuous variables were estimated by the independent *t* test (two groups) and ANOVA (more than two groups). Bonferroni’s post-hoc correction was applied. All tests were bilateral with p at 0.05. SPSS 28.0 for Windows was used to perform the statistical analyses.

## 3. Results

As shown in Table 1, the sample included 186 Caucasian FEP patients (56.5% male, mean age 30.8 SD 9.4). Regarding diagnosis, 21.5% of them were affected by schizophrenia, 57.5% by non-schizophrenic, non-affective psychosis, and 21.0% by affective psychosis. 

Nearly 59% of subjects declared at least one major stressful life event in the previous 6 months. 

The genotype distributions of the 5-HTTLPR (N = 186), rs25531 (N = 186), and COMT Val^158^Met (N = 100) polymorphisms are presented in Table 2. In addition, as previously conducted [11,36], the clustered phased haplotypes frequencies were estimated, transforming the classification into a triallelic model according to the 5-HTT allele expression levels: S_A_S_A_, L_G_S_A_, L_G_L_G_ as S′S′ (21%); L_A_S_A_ and L_A_L_G_ as L′S′ (53.2%); and L_A_L_A_ as L′L′ (25.8%). Hardy–Weinberg criteria were fulfilled for the 5-HTTLPR (SS N = 32, SL N = 97, LL N = 57; Chi-square test, *p* = 0.698) genotype distributions as well as for the triallelic model (L_A_L_A_ N = 48, L_G_L_A_/SL_A_ N = 99, L_G_L_G_/SL_G_/SS N = 48; Chi-square test, *p* = 0.977) and for COMT (Val/Val N = 27, Val/Met N = 46, Met/Met N = 27; Chi-square test, *p* = 0.726) [11]. The sub-group of 100 patients who were genotyped for both 5-HTTLPR/rs25531 and COMT did not differ from the remaining 86 patients who were genotyped only for 5-HTTLPR/rs25531 with respect to gender (Chi-square test, *p* = 1.000), life events (Chi-square test, *p* = 1.000), the 5-HTTLPR genotype (Chi-square test, *p* = 0.169), and the Hamilton total score (independent t test, *p* = 0.751). 

Polymorphism frequencies for the 5-HTTLPR and rs25531 genotypes and the clustered phased haplotype frequencies were not associated with gender (Chi-square test, *p* = 0.721, *p* = 0.078 and *p* = 0.557, respectively) nor to life events (Chi-square test, *p* = 0.114, *p* = 1.000 and *p* = 0.098, respectively). Due to frequencies < 5, no test for association with gender and life events was applied for the phased haplotype frequencies. In the sub-group of 100 patients, COMT polymorphism frequencies were not associated with gender (Chi-square test, *p* = 0.426) and life events (Chi-square test, *p* = 0.643).

To test the hypothesis that the exposure to stressful events is associated with higher depressive symptoms, two groups were defined according to the presence of at least one SLE in the previous 6 months. As shown in Table 3, higher levels of depressive symptoms were associated with SLE in the patients genotyped for 5HTTLPR (N = 186, *p* = 0.019) and in the sub-sample genotyped for COMT (N = 100, *p* = 0.009). 

To test the hypothesis that the genotype influences the severity of depression, the sample was stratified according to the genotype frequencies. As shown in Table 4, no differences in the depression scores were found for genotypes of 5-HTTPLR and rs25531 (*p* = 0.697 and *p* = 0.688, respectively) or clustered phased haplotype (*p* = 0.744). When the 5-HTTLPR genotype was taken into account, the depression score was no different among patients assuming a dominant model SS/SL vs. LL (19.7 SD 9.0 vs. 19.8 SD 8.8; independent t test, *p* = 0.952) or a recessive model SS vs. SL/LL (18.6 SD 8.5 vs. 20.0 SD 9.0; independent t test, *p* = 0.408). The same result was found regarding the clustered phased haplotype distribution assuming a dominant model L’S’/S’S’ vs. L’L’ (20.0 SD 9.1 vs. 19.1 SD 8.3; independent t test, *p* = 0.562) or a recessive model S’S’ vs. L’S’/L’L’ (19.4 SD 8.5 vs. 19.8 SD 9.0; independent *t* test, *p* = 0.760). By considering the COMT genotype, patients who were Val^158^Val showed higher levels of depression when compared to those who were Val^158^Met or Met^158^Met (*p* = 0.029). By using a dominant model for COMT Val^158^Met, it was found that patients who were Val^158^ homozygotes had higher levels of depression than individuals who were Met carriers (23.7 SD 8.5 vs. 18.5 SD 8.5; independent *t* test, *p* = 0.009), while under a recessive model, Met^158^Met vs. Val^158^Val or Val^158^Met, no difference was found (19.3 SD 7.3 vs. 20.2 SD 9.3; independent *t* test, *p* = 0.657). 

Patients were stratified for genotype and having had at least one SLE in the previous 6 months to explore a possible effect on depression. As shown in Table 5, no significant difference was found in the levels of depression for 5-HTTPLR and rs25531 (*p* = 0.091 and *p* = 0.142, respectively) or the clustered phased haplotype (*p* = 0.122). In the sub-group genotyped for COMT, a significant difference was found (*p* = 0.003). The most severe depression was shown by subjects experiencing stressful life events and were Val^158^ homozygotes, while a less severe level was found for subjects who did not have life events and were Met carriers (HAMILTON total score 25.4 SD 7.5 and 15.8 SD 7.2, respectively; Bonferroni’s post-hoc, *p* = 0.002).

## 4. Discussion

To our knowledge, this is the first study of a sample of first episode psychotic patients that showed that higher levels of depression is associated with the presence of at least one stressful life event and with COMT Val158 allele homozygosity, but not with carrying the S allele of 5-HTTLPR or of the clustered phased haplotype. Moreover, the COMT gene moderates the association between depression and stressful life events as Val^158^ allele homozygote patients experiencing stressful life events had the highest level of depressive symptoms, while a less severe level was observed in individuals who did not experience life events and were Met carriers.

The association between increased depressive symptoms and stressful life events is consistent with the reporting of the environmental factor often occurring shortly before the onset of both psychosis [17,18] and depression [19,20], contributing to both disorders by “activating’ a genetic vulnerability [21]. The novelty of the results of the present study concerns the genetic background, finding an association between depression and the COMT gene but not with the 5-HTTLPR gene. Thus, at least in psychosis, the presence of depressive symptoms is due to an altered dopamine transmission, but not to the serotoninergic one. As pointed out recently, despite the fact that the serotonin theory of depression has been very influential [37], the metanalyses [38,39,40,41] did not report convincing evidence. Specifically, while some metanalyses found an association between the S allele of 5-HTTLPR and depression [38,40], the most recent and methodologically well-conducted studies did not find this association [41,42]. Interestingly, the latter studies also excluded an interaction between serotonin and stress on depression, as found in the present study. On the other hand, in the present study, higher levels of depression were found to be associated with COMT Val158 allele homozygosity, extending previous evidence that an imbalance of dopaminergic transmission in psychosis is associated with a higher level of positive symptoms [18,43]. Finally, it was found that the COMT gene moderates the association between depression and stressful life events as Val158 allele homozygote patients experiencing stressful life events had the highest level of depressive symptoms, while a less severe level was observed in individuals who did not experience life events and were Met carriers. This result demonstrates, for the first time in the field of psychosis, that there is an interaction between genetics and environment in determining the levels of depression.

Some strengths and limitations need to be taken into account. The main strength is that the depressive level was assessed shortly after the first patient contact with treating mental health services using a validated instrument (the Hamilton scale). Thus, depressive syndrome is very close to that exhibited by patients at the illness onset, allowing one to test with as much accuracy as possible whether experiencing at least one severe stressful event in the 6 months before the onset had an impact on depression and whether there is an effect of genetic polymorphisms and SLEs on the depressive level. Second, several polymorphisms were taken into account regarding both dopamine and serotoninergic imbalance, and did not focus only on 5-HTTPLR. However, dopamine transmission is regulated by several genetic variants interacting with the COMT Val^158^Met [44,45]. Thus, taking into account only this polymorphism does not allow us to make definitive assumptions regarding the effect of this specific genetic variant on depression, both alone and in combination with SLEs. Moreover, genotype and phenotypic stratification within a small sample size of this study limited both the power and generalizability of the results. 

Despite these limitations, the present study provides initial evidence for an effect of the COMT Val^158^ homozygosity and severe stressful life events on the severity of depressive symptoms in first episode psychosis. It might be argued that the pattern characterized by the presence of Val158 allele homozygosity and SLE may be predictive of worse treatment responsiveness. Interestingly, it has been pointed out that subjects with psychosis may be predisposed to a low capacity for handling stress and thus experience more events as stressful or distressing, further increasing the sensitivity to stress [46]. It has also been found that a worse response to anti-psychotic treatment is associated with COMT Val158 allele homozygosity in schizophrenia [47]. Thus, treating subjects from the beginning of psychosis, using effective psychological treatment for stress reactivity such as Integrated-Coping Awareness Therapy (I-CAT) [48], could be useful in order to reduce depression and prevent disability. To our knowledge, the relationship between depressive symptoms in psychosis and COMT Val158 homozygosity remains poorly studied. Further replication studies in larger samples of patients are required to confirm these results.

## Figures and Tables

**Table 1:** Socio-demographic and clinical characteristics of the sample (N = 186).

Socio-Demographic and Clinical Characteristics	N (%) or Mean (SD)
Gender (Male)	105 (56.5%)
Age (years)	30.8 (9.4)
Education (2 missing)	
Primary school	86 (46.7%)
Higher school	98 (53.3%)
Living condition (4 missing)	
Alone	18 (9.9%)
With partner and/or children	49 (26.9%)
With other relatives	111 (61.0%)
With other people	4 (2.2%)
Working status (5 missing)	
Employed	83 (45.9%)
Unemployed	60 (33.1%)
Housewife/Student/Retired/Other condition	38 (21.0%)
Diagnosis	
Schizophrenia	40 (21.5%)
Non-schizophrenic, non-affective psychosis	107 (57.5%)
Affective psychosis	39 (21.0%)
PANSS total score (1 missing)	
Positive symptoms	20.8 (7.0)
Negative symptoms	17.9 (9.5)
General symptoms	43.0 (13.3)
Total symptoms	81.8 (23.0)
HAMILTON total score	19.7 (8.9)
Life events	
At least one major stressful event in the 6 months preceding illness onset	109 (58.6)

**Table 2:** Genotype frequencies, phased and clustered phased haplotype frequencies for 5-HTTLPR and rs25531 (N = 186), and genotype frequencies for COMT rs 4680 (N = 100).

Polymorphism Frequencies	N (%)
5-HTTLPR genotype (N = 186)	
SS	32 (17.2%)
LS	97 (52.2%)
LL	57 (30.6%)
rs25531 genotype (N = 186)	(1 missing)
AA	168 (90.8%)
AG	17 (9.2%)
GG	0 (0%)
**Phased haplotype frequencies (N = 186)**	
L_A_L_A_	48 (25.8%)
L_G_L_A_	8 (4.3%)
L_A_S_A_	89 (47.8%)
L_A_S_G_	2 (1.1%)
L_G_L_G_	1 (0.5%)
S_A_L_G_	6 (3.2%)
S_A_S_A_	31 (16.7%)
S_A_S_G_	1 (0.5%)
**Clustered phased haplotype frequencies (N = 186)**	
S’S’	39 (21.0%)
S’L’	99 (53.2%)
L’L’	48 (25.8%)
**Polymorphism frequencies**	N (%)
COMT rs 4680 (N = 100)	
Val/Val	27 (27.0%)
Val/Met	46 (46.0%)
Met/Met	27 (27.0%)

**Table 3:** Mean score differences in depression (HAMILTON) between patients who experienced at least one major stressful event in the 6 months preceding illness onset and patients who did not.

	Major Stressful Events in the 6 Months Preceding Illness Onset		*p*-Value *t* Test
	No Event	At Least One Event	
**Pts. with 5HTTLPR genotype (N = 186)**			
HAMILTON total score	17.9 (8.4)	21.0 (9.0)	0.019
**Pts. with COMT genotype (N = 100)**			
HAMILTON total score	17.2 (8.1)	21.8 (8.7)	0.009

**Table 4:** Mean score differences in depression (HAMILTON) among the 5HTTLPR, rs25531, the clustered phased haplotype, and the COMT genotypes.

	5HTTLPR Genotype			*p*-Value ANOVA
	SS	LS	LL	
**Pts. with 5HTTLPR genotype (N = 186)**				
HAMILTON total score	18.6 (8.5)	20.1 (9.1)	19.8 (8.8)	0.697
	**rs25531 genotype**			
	**AA**	**GA/AG**		***p*-value** ***t* test**
HAMILTON total score	19.6 (8.7)	20.5 (10.5)		0.688
	**Clustered phased haplotype genotype**			
	**SS**	**LS**	**LL**	***p*-value** **ANOVA**
HAMILTON total score	19.4 (8.5)	20.2 (9.4)	19.1 (8.3)	0.744
	**COMT genotype**			***p*-value ANOVA**
	**Val/Val**	**Val/Met**	**Met/Met**	
**Pts. with COMT genotype (N = 100)**				
HAMILTON total score	23.7 (8.5)	18.1 (9.1)	19.3 (7.3)	0.029

**Table 5:** Effect of major stressful events in the 6 months preceding illness onset and 5HTTLPR and COMT genotypes on the depression (HAMILTON) mean scores.

	No Event SS (N = 11)	No Event LS/LL (N = 66)	At Least One Event SS (N = 21)	At Least One Event LS/LL (N = 88)	*p*-Value ANOVA	Bonferroni’s Post-Hoc
Pts. with 5HTTLPR genotype (N = 186)						
HAMILTON total score	16.7 (7.1)	18.1 (8.6)	19.5 (9.2)	21.4 (9.0)	0.091	-
	**No event** **AA (N = 70)**	**No event** **AG/GA (N = 7)**	**At least one event** **AA (N = 98)**	**At least one event** **AG/GA (N = 10)**	***p*-value** **ANOVA**	**Bonferroni’s post-hoc**
**Pts. with rs25531 genotype** **(N = 186)**						
HAMILTON total score	17.9 (7.8)	18.1 (13.7)	20.8 (9.2)	22.2 (8.0)	0.142	-
	**No event** **SS (N = 13)**	**No event** **LS/LL (N = 64)**	**At least one event** **SS (N = 26)**	**At least one event** **LS/LL (N = 83)**	***p*-value** **ANOVA**	**Bonferroni’s post-hoc**
**Pts. with haplotype genotype** **(N = 186)**						
HAMILTON total score	16.8 (6.6)	18.2 (8.7)	20.6 (9.1)	21.2 (9.1)	0.122	-
	**No event** **Val/Val (N = 11)**	**No event** **Val/Met//Met/Met (N = 30)**	**At least one event** **Val/Val (N = 16)**	**At least one event** **Val/Met//Met/Met (N = 43)**	***p*-value** **ANOVA**	**Bonferroni’s post-hoc**
**Pts. with COMT genotype** **(N = 100)**						
HAMILTON total score	21.1 (9.6)	15.8 (7.2) ^a^	25.4 (7.5) ^a^	20.5 (8.9)	0.003	^a^*p* = 0.002

## Data Availability

The data supporting the results are not publicly available, but can be provided by the corresponding author [ST] on reasonable request.
